# Supplementary material for: Balanced Viscoelastic Properties of Pressure Sensitive Adhesives Made with Thermoplastic Polyurethanes Blends
Source: Polymers (Basel). 2019 Oct 3;11(10):1608. doi: 10.3390/polym11101608 (PMC6835935; doi:10.3390/polym11101608)
Supplement: Supplementary File 1 [file polymers-11-01608-s001.pdf]

polymers

Supplementary Material:

Balanced Viscoelastic Properties of Pressure Sensitive Adhesives Made with Thermoplastic Polyurethane Blends

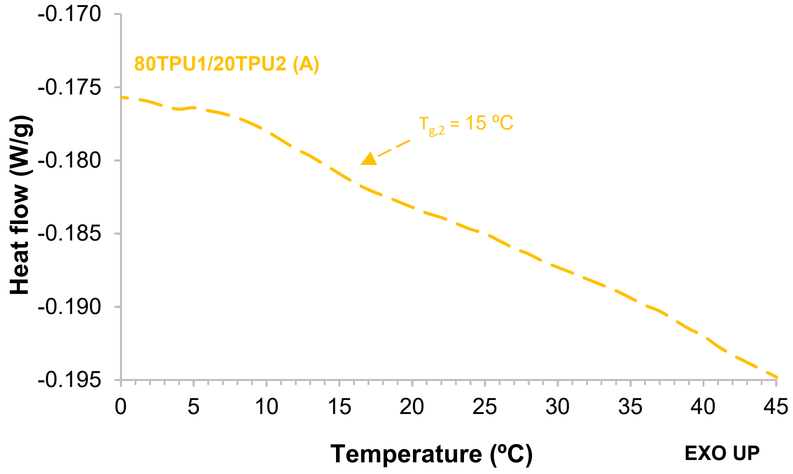

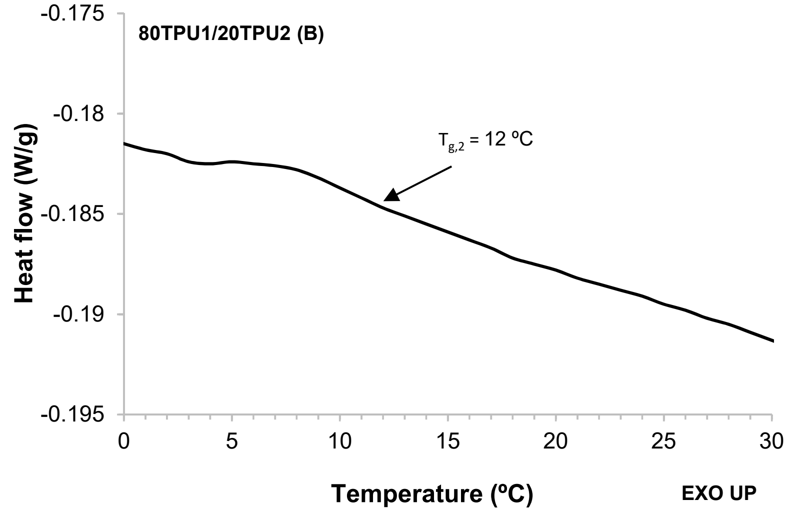

Figure S1. Expanded region of the DSC thermograms of 80TPU1/20TPU2 (**A**) and 80TPU1/20TPU2 (**B**) blends showing more clearly the glass transition due to the soft segments. Second DSC heating run.

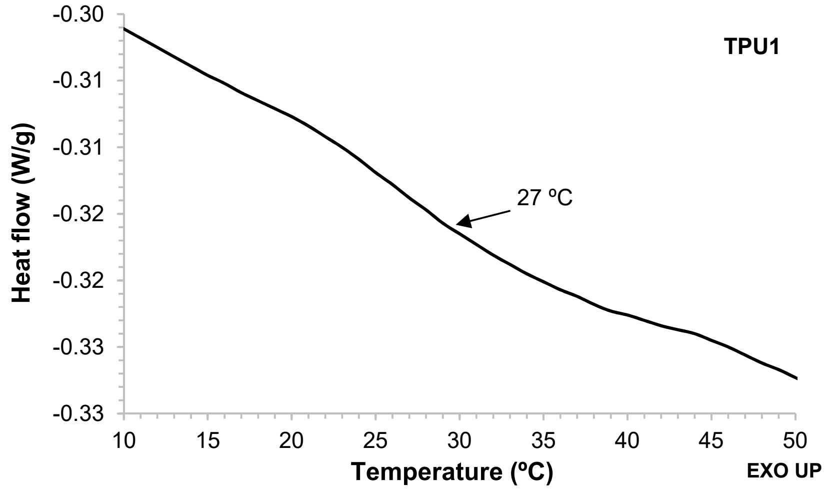
TPU1
Heat flow (W/g)
-0.30
-0.31
-0.31
-0.32
-0.32
-0.33
-0.33
27 ºC
10
15
20
25
30
35
40
45
50
Temperature (ºC)
EXO UP

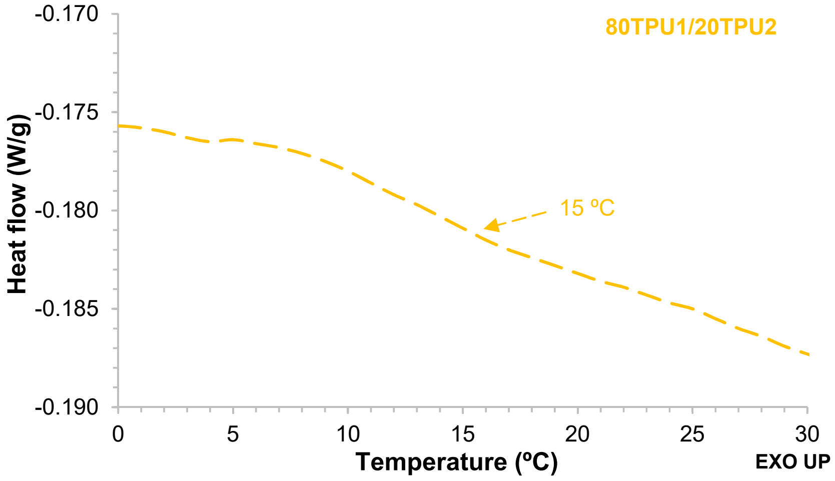
80TPU1/20TPU2
Heat flow (W/g)
-0.170
-0.175
-0.180
-0.185
-0.190
15 ºC
0
5
10
15
20
25
30
Temperature (ºC)
EXO UP

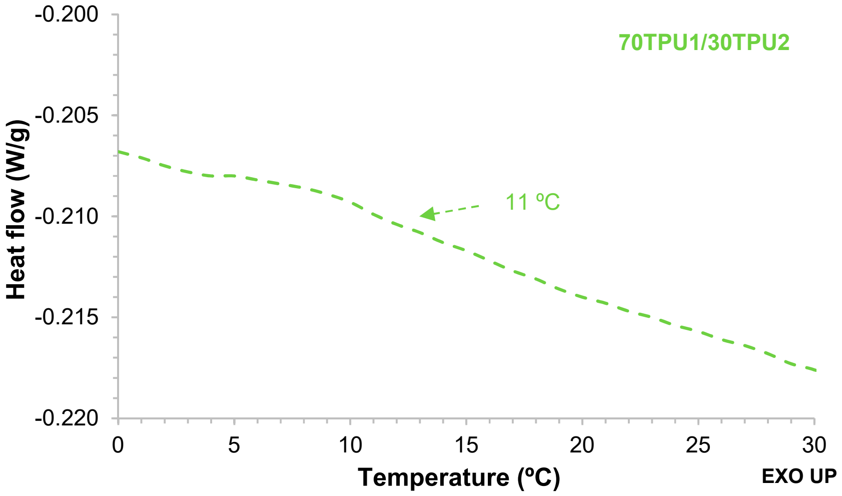
70TPU1/30TPU2
Heat flow (W/g)
-0.200
-0.205
-0.210
-0.215
-0.220
11 ºC
0
5
10
15
20
25
30
Temperature (ºC)
EXO UP

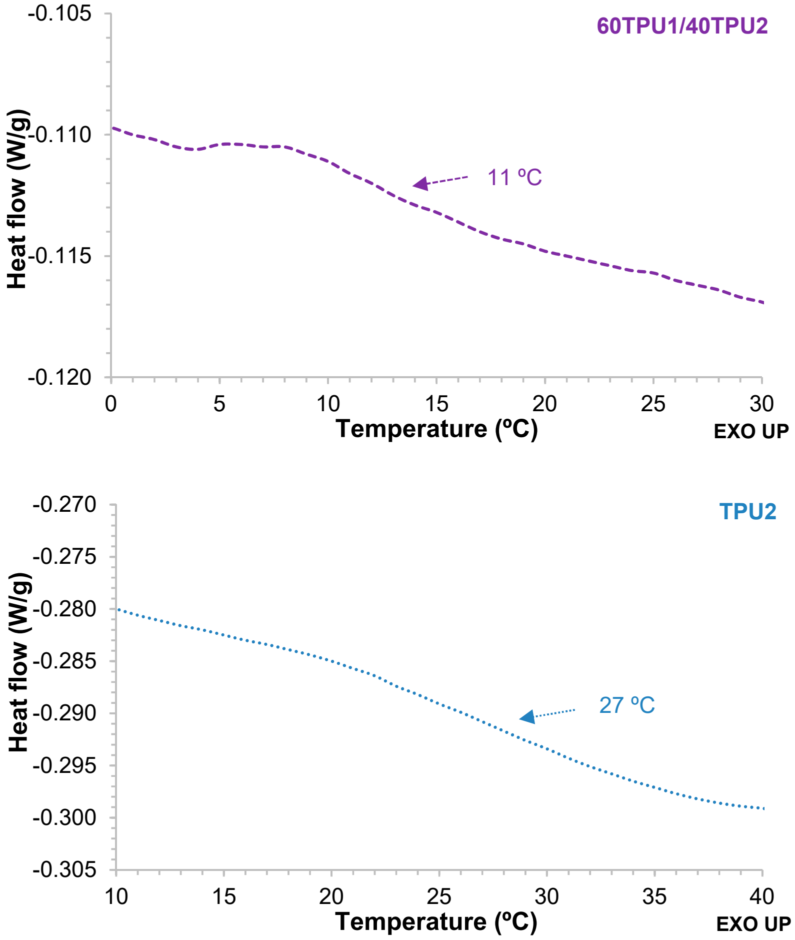

Figure S2. Expanded region of the DSC thermograms of TPU1, TPU2, and TPU1 + TPU2 blends, showing more clearly the glass transitions due to the soft segments. Second DSC heating run.

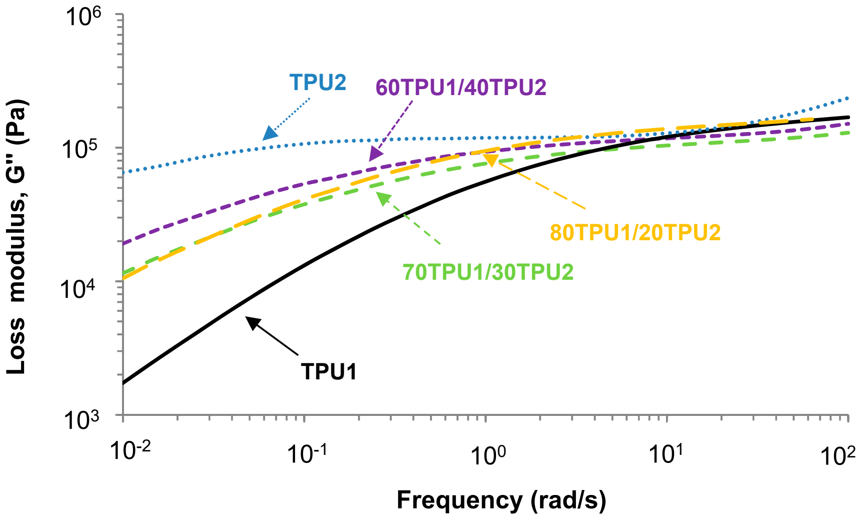

Figure S3. Variation in the loss modulus (*G*′) at 25 °C as a function of the frequency of TPU1, TPU2, and TPU1 + TPU2 blends. Plate-plate rheology experiments. Frequency sweep.
